# Microalgae *Chlorella* Species as Biofertilizer: Towards Sustainable Crop Nutrition and Environmental Benefits

**DOI:** 10.3390/biology15131062

**Published:** 2026-07-03

**Authors:** Mounia Chroho, Eleftherios Touloupakis, Cecilia Faraloni, Latifa Bouissane

**Affiliations:** 1 Sustainable Processes, Advanced Materials and Computational Chemistry Team, Polydisciplinary Faculty of Beni Mellal, Sultan Moulay Slimane University, P.O. Box 592 Mghila, Beni Mellal 23000, Morocco; mounia.chroho@usms.ma; 2 Istituto di Ricerca sugli Ecosistemi Terrestri, CNR, Via Madonna del Piano 10, 50019 Sesto Fiorentino, Italy; eleftherios.touloupakis@cnr.it; 3 Istituto per la Bioeconomia, CNR, Via Madonna del Piano 10, 50019 Sesto Fiorentino, Italy

**Keywords:** Microalgae, *Chlorella*, biofertilizers, wastewater treatment

## Abstract

Chemical fertilizers are widely used to achieve rapid and high-yield crop production. However, their intensive use negatively impacts ecosystems by polluting air and groundwater, accelerating soil acidification and deterioration, and because they rely on energy-intensive production processes and excessive mining. Biofertilizers have emerged as a promising and sustainable solution. Among them, microalgae, particularly species of the genus *Chlorella*, have attracted significant attention. *Chlorella* microalgae are an eco-friendly and cost-effective biofertilizer option, with advantages such as ease of cultivation, fast growth and slow release of nutrients when applied to soil. In this context, this review summarizes the potential of *Chlorella* species as biofertilizers and highlights the important role of its phytohormones in this effect, in addition to its use in wastewater treatment, which results in biomass and water with biofertilizing potential.

## Simple Summary

Chemical fertilizers are costly and bring multiple environmental concerns: they contribute to serious ecological issues, including air and groundwater pollution, soil acidification and deterioration, and root damage. Biofertilizers constitute eco-friendly alternatives to conventional chemical fertilizers, and can boost high-yield crop production and improve root development. Microalgae constitute a promising solution and are being increasingly recognized for their high potential as biofertilizers, capable of restoring soil fertility, promoting plant growth, and enhancing the chemical and biological characteristics of soil. Among the microalgae species studied for their economic potential, *Chlorella* stands out as particularly promising for biofertilizer production after several studies in Petri dishes and in soil. *Chlorella* is also used to treat wastewater and the resulting biomass and water have considerable biofertilizing effects.

## 1. Introduction

Microalgae are unicellular, photosynthetic microorganisms that inhabit a variety of habitats. They have a simple cellular structure and their aquatic environment provides abundant access to water, CO_2_, and other essential nutrients, making them highly effective at converting solar energy into biomass [1].

Microalgal biomass is of great value due to its wide range of biotechnological applications and its ability to produce by-products that may help address major global issues, such as the energy crisis and the depletion of fossil fuel reserves. With the growing need to identify alternative energy sources with low greenhouse gas emissions [2], microalgae have emerged as key candidates for the development of biofuels, bioenergy, biopolymers and biopesticides [3,4,5]. They also contain a variety of bioactive compounds with applications in nutrition, cosmetics, and medicine [6,7,8,9].

In modern agriculture, microalgae are being increasingly recognized for their high potential as biofertilizers, capable of restoring soil fertility, promoting plant growth, and enhancing the chemical and biological characteristics of soil [10,11,12,13]. Biofertilizers constitute an eco-friendly alternative to conventional chemical fertilizers, which are widely used to boost high-yield crop production. They foster agricultural productivity by improving root development, nutrient uptake, and plant resilience to diseases, pests, frost and drought [14].

Seventeen essential nutrients are required for optimal plant growth, with potassium (K), phosphorus (P), and nitrogen (N) being the primary macronutrients [15]. Nitrogen and phosphorus fertilizers are typically produced through chemical processes using air and phosphate rock as basic feedstocks. However, the production of nitrogenous fertilizers is particularly energy intensive; it is the fourth-largest energy consumer in the US chemical production industry and the second-largest globally [15]. Around 70% of global phosphate rock is mined in Morocco and the Sahara region [16], creating vulnerabilities in the supply chain due to the uneven distribution, mining waste, land use issues and increasing production costs, all of which affect the economic viability of agricultural products [17].

In 2019, global consumption of phosphorus and nitrogen fertilizers reached 71 (as P_2_O_5_) and 132 tons, respectively. As the demand for food and feed continues to rise, the need for these fertilizers is expected to increase [15]. However, their widespread use brings multiple environmental concerns. Chemical fertilizers are costly and contribute to serious ecological issues, including air and groundwater pollution, soil acidification and deterioration, and root damage, ultimately increasing plant vulnerability to infection. These factors further exacerbate food insecurity and greenhouse gas emissions [18,19]. Some countries have taken action to address these issues. China, which accounts for over 30% of the global fertilizer consumption and is currently the world’s largest fertilizer user [19], has introduced policies to reduce its reliance on chemical inputs [20,21].

In this context, interest in eco-friendly, cost-effective alternatives such as biofertilizers is increasing. Microalgae constitute a promising solution to mitigate the harmful environmental impacts of the continued use of chemical fertilizers [22,23]. They can act as organic fertilizers and offer several advantages, including the slow release of N, P, and K, which meets plant needs while preventing nutrient losses [1]. Among the microalgae species studied for their economic potential, *Chlorella* stands out as particularly promising for biofertilizer production [24,25]. The first species of *Chlorella* to be discovered was *Chlorella vulgaris*, identified by Beijerinck in 1890. Since then, many other coccoid green algae have been discovered and categorized as belonging to the genus *Chlorella* [26]. Hundreds of *Chlorella* species have been reported in the literature [27,28,29], but the taxonomy of the genus remains confusing and problematic. Initial classifications were based on morphological features, even though *Chlorella* species lack clear distinguishing features and show variation from species to species [28]. Later, taxonomic approaches shifted to an ultrastructural concept and, more recently, to molecular strategies based on DNA and RNA analyses [26]. Currently, integrative or polyphasic techniques are recommended. These combine traditional and contemporary techniques, such as phylogenetic analysis of distinctive gene sequences, along with life-cycle and biochemical marker investigations. As a result of the development of these taxonomic methods, many species initially assigned to the *Chlorella* genus have been reclassified into other genera.

*Chlorella* species are distributed among two classes of chlorophytes: Trebouxiophyceae and Chlorophyceae [26,28]. In 1996, nine species were recorded within the genus, seven belonging to Trebouxiophyceae (*C. vulgaris*, *C. sorokiniana*, *C. minutissima*, *C. ellipsoidea, C. luteoviridis*, *C. mirabilis*, and *C. saccharophila*), and two to Chlorophyceae (*C. zofingiensis* and *C. fusca*). Subsequent studies that revisited the confused classification of *Chlorella* concluded that only five, four or even three “true” *Chlorella* species may exist, including *C. vulgaris*, *C. lobophora*, and *C. sorokiniana* [28].

*Chlorella* biomass has emerged as a promising sustainable input in agriculture, combining the functions of a biofertilizer and a biostimulant. Rich in essential nutrients, *Chlorella* biomass contributes directly to soil fertility and plant nutrition when applied to crops. At the same time, it exhibits strong biostimulant properties due to the presence of bioactive compounds that enhance plant metabolic activity, improve nutrient uptake efficiency, and promote stress tolerance. This dual functionality allows *Chlorella*-derived products to support plant growth not only by supplying nutrients, but also by stimulating physiological processes, ultimately leading to improved crop productivity and resilience under both optimal and adverse environmental conditions.

Many species of the genus *Chlorella* have been investigated for biofertilizer applications because of their high growth rates, metabolic versatility, and ability to produce a wide range of bioactive compounds [30]. The most studied species include *Chlorella vulgaris, C. sorokiniana, C. zofingiensis*, and *C. pyrenoidosa*, each exhibiting specific physiological traits that make them suitable for agricultural use [31]. These microalgae are characterized by rapid biomass accumulation and high photosynthetic efficiency, enabling cost-effective large-scale cultivation. *Chlorella* can accumulate significant amounts of macro- and micronutrients and produce plant growth-promoting substances. Furthermore, *Chlorella* species show remarkable tolerance to varying environmental conditions, including temperature fluctuations and nutrient stress, making them adaptable to diverse cultivation systems, including wastewater-based production. Compared with other microalgae, *Chlorella* combines ease of cultivation, high biomass productivity, and well-documented biostimulant effects, which explains its widespread use in biofertilization studies.

The aim of this review is to summarize the research on the biofertilizing and biostimulating effects of *Chlorella* species. Since most of the available studies focus on laboratory or greenhouse experiments, compiling these data could aid in decision making regarding the direct application of *Chlorella* in large-scale agriculture. Species names are presented as they appear in the references, even if the name has since been changed and assigned to another genus.

## 2. Research Methodology

A literature search was performed in the Scopus, PubMed, and Google Scholar web search engines using the keywords “Algae”, “*Chlorella*”, and “Biofertilizers”. Only studies published from 2014 onward were included. Only articles published in English were considered for further analysis.

## 3. Biomass Composition Extraction and Preparation Methods

The composition of a residue composed of *Chlorella pyrenoidosa, Chlorella vulgaris, Chlorella ellipsoidea*, and *Chlorella regularis* was characterized in a recent study and was found to contain a broad spectrum of nutrients [32]. The concentrations of key macronutrients such as total nitrogen (97,910 mg/kg), total phosphorus (11,760 mg/kg), and total potassium (5500 mg/kg) were determined. Inorganic nitrogen forms such as nitrate and ammonium were present at relatively low levels, indicating that the majority of nitrogen was in an organic form. Total magnesium (4300 mg/kg) and total calcium (9700 mg/kg) were also measured. The biomass was rich in micronutrients, including iron and manganese, and the low C/N ratio (5.1) supports the suitability of *Chlorella* as an effective source of organic nitrogen. Overall, the presence of substantial amounts of secondary macronutrients and micronutrients highlights its potential value as a nutrient-rich agricultural input. Wichaphian et al., in their recent study, present a detailed description of the biomass composition of *Chlorella pyrenoidosa*, which was then used as a biofertilizer [33]. The biomass contained 6.69% nitrogen, 4.31% phosphorus, 0.18% potassium, 0.52% calcium, 0.34% magnesium, and 72.78% organic matter.

The effectiveness of *Chlorella*-derived biomass as a biofertilizer and biostimulant depends strongly on how the biomass is processed before application [34]. The extraction and treatment methods play a crucial role in determining the availability, integrity, and activity of bioactive compounds. Factors such as the extraction process, application method, crop type, and dosage significantly influence biostimulant performance. There are many extraction methods including aqueous extraction, ultrasonication, freezing and thawing, microwave-assisted extraction, enzymatic lysis, osmotic shock, and the use of organic solvents [35,36]. These methods can yield products with markedly different compositions and biological effects; in some cases, inappropriate processing or excessive concentrations may even induce phytotoxic responses [34]. For this reason, current research is increasingly focused on developing mild and sustainable extraction techniques that avoid the use of toxic solvents and harsh conditions, which could otherwise degrade sensitive bioactive molecules within algal cells.

While biomass composition and extraction processes are key factors determining the biochemical profile of *Chlorella*-derived products, methodological aspects related to biomass production, harvesting, and application are equally important for correctly interpreting their performance as biofertilizers. *Chlorella* cultivation is a key step that strongly determines biomass productivity, biochemical composition, and ultimately the effectiveness of derived biofertilizers. Microalgal cultivation systems are broadly classified into open and closed bioreactors. Open bioreactors, such as raceway ponds, are widely employed due to their low capital and operational costs and relative ease of scale-up. However, they present disadvantages such as susceptibility to contamination, evaporation, and environmental variability, which can lead to fluctuations in biomass yield and quality. In contrast, closed bioreactors provide a higher level of control over cultivation parameters such as light intensity, temperature, and CO_2_ supply, thereby enhancing productivity and reproducibility, but at the expense of higher capital costs and increased energy and infrastructure requirements [31,37]. Another important factor is the selection of culture conditions, including growth medium composition, light intensity, temperature, and pH, which significantly influence not only biomass yield, but also its biochemical profile, including the accumulation of bioactive compounds. These variations are particularly relevant for agricultural applications, as they may directly affect biofertilizer performance and consistency across different production systems.

*Chlorella* biomass can be applied either as harvested material, following separation from the growth medium by centrifugation, filtration, or flocculation, or as part of the whole culture without any prior processing [31]. Although the direct application of untreated cultures offers practical advantages in terms of cost-effectiveness and ease of use, it also introduces potential variability due to the presence of residual growth medium. In contrast, the use of washed or concentrated biomass allows for a more controlled assessment of the specific effects of *Chlorella*, albeit at the expense of additional processing steps that may limit scalability. Pre-treatment strategies, including drying, cell disruption, and extraction, can alter the availability of bioactive compounds such as phytohormones, amino acids, and peptides, thereby affecting the overall efficacy of *Chlorella*-based products. The wide variability in preparation and application methods across studies highlights the need for greater methodological standardization to enable more reliable comparisons and to better elucidate the mechanisms underlying microalgal biofertilization.

## 4. Effects of *Chlorella* on Plant Growth

*Chlorella* species have shown encouraging potential in a range of agricultural applications. Among them, *Chlorella vulgaris* is the most widely tested species, and its effects have been addressed in terms of seed germination, plant growth and both the quantity and quality of the obtained fruits. When applied as a suspension to Petri dishes containing tomato seeds in a sterilized culture medium, *C. vulgaris* accelerated seed growth compared to the control (sterilized culture medium). A suspension with 0.17 g/L dry biomass increased root and shoot lengths by 100% and 87.9%, respectively [38]. For cucumber, a *C. vulgaris* suspension at a concentration of 0.25 g/L resulted in shoot and root lengths that were 2.1 and 2.4 times higher, respectively, than those of the control group [38]. Similar positive effects were observed in the germination of barley and wheat grains, which were enhanced by a *Chlorella* suspension. The optimal concentrations for promoting root and shoot lengths in barley and wheat seeds were 0.06 g/L and 0.23 g/L, respectively [39].

**Figure 1:**
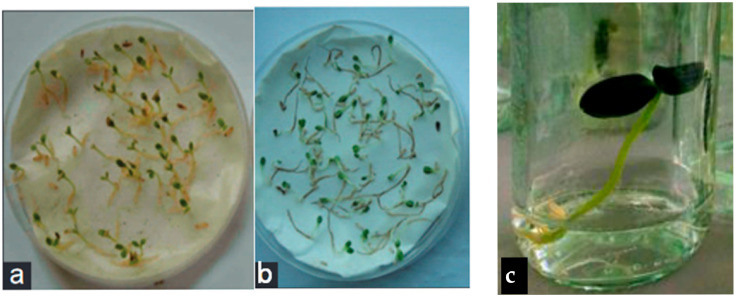
Representative example of plant responses to *Chlorella* application. (**a**) Four-day-old seedlings at the beginning of water stress; (**b**) treated lettuce seedlings after 7 days of water stress; (**c**) hydroponic culture in *C. vulgaris* suspension. Adapted from Elhafiz et al. [40] and Vildanova et al. [41], both licensed under CC BY.

The effects of *C. vulgaris* and *C. pyrenoidosa* were also tested on cucumber, lettuce, eggplant, and rice seeds. The seeds were incubated in Petri plates on Whatman filter paper and were watered twice daily with 2 mL of either a *C. vulgaris* or *C. pyrenoidosa* solution. Seedlings treated with these solutions exhibited healthier and more developed root systems and greener leaves compared to the control. For example, lettuce seedlings watered with the *Chlorella* solution remained viable for a full week, whereas those in the control group dried out in three to four days (Figure 1a,b) [40].

*C. vulgaris* suspensions have also shown beneficial effects in hydroponic systems. In cucumber cultures, application of *C. vulgaris* increased the plants’ dry biomass (Figure 1c) and has been recommended for use in hydroponic plant cultivation [41].

Chabili et al. studied the effect of extracted components from *Chlorella vulgaris*, obtained using four extraction techniques, on the germination performance of wheat and tomato seeds [34]. Wichaphian et al. investigated the effects of *Chlorella* biomass on lettuce seedlings, assessing plant responses through measuring physiological parameters, biochemical properties, and rhizosphere microbial dynamics. The results showed that *Chlorella* biomass enhanced root development by 34.41% and increased the chlorophyll content by 39.05%, supporting its potential as an effective biofertilizer [35].

Chabili et al. evaluated the effects of extracts of *Chlorella* sp. GA18 and *Chlorella* sp. GA65, among other soil microalgae, on wheat growth, physiology, yield, and quality under controlled conditions [42]. They observed improvements in growth parameters, physiological traits, and yield components.

In addition to studies conducted under laboratory conditions, the biofertilizing effects of *Chlorella* species have also been demonstrated in soil-based pots. These studies further confirmed the benefits of using *Chlorella* spp. in agricultural settings. For example, in soil pot experiments with *Hibiscus esculentus*, the use of *Chlorella* significantly reduced germination time and resulted in greater plant height and higher fruit counts [14]. For *Zea mays* cultivated in soil inoculated with *C. oocystoides* and *C. minutissima*, the research team observed a marked increase in biomass accumulation and in chlorophyll, potassium, and phosphorus contents [43]. The application of an aqueous extract from *C. sorokiniana*, obtained after harvesting the biomass from an algal growth medium, was tested on *Triticum aestivum* var. CAMARGO (wheat seeds). The results showed a 30% increase in plant length, with improvements of 22% and 51% in the total dry biomass of aboveground and belowground components, respectively [17].

In another study, the strawberry variety ‘Kuemsil’ planted in soil and irrigated with a *C. fusca* solution (1.0 × 10^7^ cells/mL) exhibited enhanced chlorophyll content, fresh weight, and phosphorus concentration. The fruit also showed improvements in quality, hardness, fresh weight and phosphorus, potassium and sugar contents [44].

Solid formulations of microalgae-based products include granules, microgranules, wettable powders, and water-dispersible granules. Notably, a granular formulation derived from dried *Chlorella vulgaris* Beijerinck, applied at doses of 2 and 3 g dry algae/kg soil, significantly enhanced soil fertility and increased the fresh and dry weights of lettuce by up to tenfold compared with the control [45].

In short, the findings to date support that the positive effects of *Chlorella* species on root, shoot, and fruit development are closely related to their influence on the plant rhizosphere and soil properties. *Chlorella* inoculation has been shown to alter key soil characteristics, such as total electric conductivity; nitrogen, potassium and phosphorus contents; soil pH; organic matter content; and organic carbon levels [14,43]. For example, soil inoculation with *C. oocystoides* at ratios of 1%, 2.5% and 5% led to increased electric conductivity, and nitrogen and organic matter contents, while significantly decreasing soil pH [43]. Treatments with *C. minutissima* also increased nitrogen and organic matter contents but decreased electrical conductivity without significantly affecting soil pH [43]. Furthermore, distinct treatments using *C. vulgaris* significantly increased the phosphorus, nitrogen and potassium contents, in addition to organic carbon and organic matter contents in the tested soils [14]. Table 1 summarizes some studies on the effect of *Chlorella* species on plant and soil properties.

Field evidence supporting the agronomic potential of microalgae has been reported. For instance, Ma et al. (2024) showed that the application of microalgae-based biofertilizers in a hawthorn orchard increased fruit yield by up to ~30% under field conditions [46]. In addition, greenhouse studies have demonstrated that *Chlorella vulgaris* can enhance plant growth, biomass accumulation, and nutrient content, supporting its potential under semi-controlled agronomic conditions [47]. Applications in crop systems such as maize have also shown significant improvements in plant growth and yield when *Chlorella*-based biofertilizers are applied, indicating their potential at larger scales [48].

**Table 1:** Comparison of the use of biomass from various *Chlorella* species across different target crops, including application methods and their effects on plant growth parameters and soil properties.

*Chlorella* Species	Target Crop or Plant	Application Type	Effect on Plant Growth Parameters	Effect on Soil Properties	Reference
*Chlorella vulgaris*	Rice and *Pennisetum x sinese*	Biofertilizer, weekly soil inoculation (from 5 to 20 mL of suspension)	Increased shoot length and soil macroaggregate proportions (fractions over 2 mm are increased by 33.62% for rice, and by 21.95% for Pennisetum)	Nutrient enrichment, soil conditioning, and regulation of Extracellular Polymeric Substances (EPSs) to enhance aggregate stability	[49]
*Chlorella vulgaris*	Tomato (*Solanum lycopersicum*/*Lycopersicon esculentum*)	Foliar spray and soil drench	Fruit length, weight, diameter, mineral content (P, Ca, K, and Mg), and root length are increased	---	[50]
*Chlorella* sp.	Rice	Water inoculation (concentration of 1.3 × 10^7^ cells/mL)	Grain yield is increased by 46.06%; effective panicle number is 55.00% higher	NH_4_^+^-N increased by 50.00–57.16%, and NO_3_^−^-N increased by 57.61–104.57%, pH decreased by 1.01–1.02%	[51]
*Chlorella sorokiniana*	Tomato (*Solanum lycopersicum*)	Root irrigation (1 L of bacterial solution diluted at a 1:9 volume ratio)	Increased plant height (11.72%), stem diameter (9.78%), and chlorophyll content	Reduced electrical conductivity by 53.68%, increased the content of soil organic carbon by 19.39%, boosted urease/protease activities	[52]
*Chlorella pyrenoidosa*	Lettuce (*Lactuca sativa*)	Soil amendment (0.5 g/kg potting soil)	Increase in shoot length (31.89%), root length (27.98%), fresh weight (47.33%), and chlorophyll content	Rapid nutrient supply (nitrogen mineralization)	[33]
*Chlorella vulgaris*	Soybean (*Glycine max* L.)	Liquid extract treatment (concentrations of 0.1, 0.5, and 2.0 g/L)	Promoted root formation by 220% (at 0.5 g/L) and 493% (at 2.0 g/L)	---	[53]
	Watercress Seeds (*Lepidium sativum* L.)		Germination index increased by 3.5% (at 0.1 g/L)		
*Chlorella vulgaris*	Wheat (*Triticum aestivum* L.)		Increased chlorophyll content by 7.5–8.0%		
*Chlorella fusca*	Melon (*Cucumis melo*)	Foliar spray	Suppression of leaf drop; increased fresh weight (1.8 kg vs. 1.6 kg); improved hardness; higher total polyphenol and free sugar contents	Enhanced photosynthetic activity, inducing greater assimilate contents	[54]

## 5. Origin of the Biofertilizing Effect of *Chlorella* Species

The use of *Chlorella* species as biofertilizer has a significant impact on plant growth, from seed germination to fruit maturation, as proven by several studies and discussed in the previous sections. The beneficial effects of *Chlorella* as a biofertilizer are attributed to its primary and secondary metabolites. *Chlorella* synthesizes proteins, carbohydrates and lipids as primary metabolites and a range of secondary metabolites that includes phytohormones, phenolics, vitamins, terpenoids and polyamines [53,55]. These metabolites can significantly increase productivity through a variety of biological actions.

Among these metabolites, phytohormones (plant hormones) are naturally occurring organic molecules that are vital for micro- and macroalgal development and functioning. In algae, these hormones include auxins, gibberellins, and cytokinins, and they are generally present in trace amounts. However, they have noticeable effects on algae that result in a range of agronomic advantages [53,56]. Their low concentration makes their extraction and identification challenging [56].

Generally, auxins are known for controlling root growth and development; gibberellins are crucial for initiating seed germination, stem elongation, and stress tolerance; and cytokinins are responsible for delaying aging, promoting organ formation, and stimulating cell division [56,57].

Extracting, analyzing, and identifying phytohormones is complicated because of their low contents, and most extraction techniques require a high volume of solvents. All this has led to the publication of very few studies on the hormones secreted by *Chlorella*. Among the identified hormones in *Chlorella* species, auxins are the most commonly extracted and studied, especially indole-3-acetic acid (IAA), the most common and physiologically active auxin that controls plant growth and development [58]. Table 2 summarizes the confirmed hormones in *Chlorella* species.

The biofertilizing effect of *Chlorella* is also related to its capacity to boost the synthesis of chlorophyll a and b [59], and to prevent nutrient loss in the soil by releasing nutrients and minerals due to its richness in macro- and micronutrients, including nitrogen, phosphorus, potassium, and magnesium, which is the central ion in the photosynthetic process [53]. In addition to supplying minerals, a recent study has demonstrated the capacity of *Chlorella vulgaris* auxin IAA as a phosphate solubilizer [58].

**Table 2:** Confirmed hormones in *Chlorella* species.

*Chlorella* Species	Phytohormone Type	Reference(s)
*Chlorella vulgaris*	Auxins, IAA	[58,60]
	IAA, jasmonic acid, salicylic acid	[61]
	Cytokinins	[56]
	IAA, gibberellic acid, and zeatin	[62]
*Chlorella sorokiniana*	IAA	[63]
	IAA, 2-oxindole-3-acetic acid and salicylic acid	[55]
*Chlorella pyrenoidosa*	IAA	[64]
*Chlorella variabilis*	Cytokinins	[65]
*Chlorella minutissima*	Auxins, cytokinins, gibberellins, abscisic acid, ethylene and brassinosteroids	[66]

## 6. *Chlorella* for Wastewater Treatment: Biofertilizing Potential of Biomass and Water

*Chlorella* is widely used in the treatment of sewage and urban wastewater due to its ability to absorb nutrients like P and N, which it converts into biomass and valuable chemicals (e.g., pigments and lipids) [67]. Both the resulting biomass and the treated wastewater have biofertilizing potential. After microalgal cultivation, treated wastewater can be reused to irrigate plants in meadows, gardens and terraces, contributing to plant nutrition. However, although wastewater supports *Chlorella* growth due to its high nutrient content, the safety of the resulting biomass cannot be inferred from nutrient availability alone. Contaminants such as pathogens, heavy metals, salinity, and emerging pollutants may accumulate in the microalgae and subsequently be transferred to soils and plants when the biomass is applied as a biofertilizer or biostimulant. Therefore, rigorous monitoring and strict compliance with regulatory frameworks (e.g., EU Regulation 2020/741) are essential to ensure the safe and sustainable use of both the biomass and reclaimed water.

*C. vulgaris* is particularly effective in wastewater treatment; it can remove or reduce harmful contaminants, especially P and N, nutrients that are often only partially eliminated by conventional wastewater treatments [68]. Under optimal light, temperature, and nutrient conditions, *C. vulgaris* cultivation in sewage increases biomass and lipid yields by using the nutrients present in the wastewater. At the same time, several key water quality parameters are significantly improved, with reported reductions in chemical oxygen demand (COD), biological oxygen demand (BOD), and ammonia, chloride, nitrate-nitrogen, phosphate, and sulfate levels [24,68]. These reductions can exceed 90% [24,69], and in some cases, complete (100%) removal of total N and P has been reported in systems involving *C. vulgaris* and *C. protothecoides* [70].

Once microalgae are fully grown and are harvested, the treated wastewater has been shown to be effective when reused as a biofertilizer. For instance, it has been used to irrigate tomato plants, resulting in increases in both fruit weight and number [24,68]. One study reported an 118% increase in tomato fruit weight [68]. Similarly, a treatment system using *C. sorokiniana* to treat municipal wastewater showed that the resulting effluent could be safely repurposed for irrigation [71].

The reuse of wastewater treated with *C. vulgaris* was also tested for irrigating wheat (*T. aestivum* L.), and the results showed improvements in shoot biomass as well as protein and carbohydrate contents [72]. In addition to the reuse of treated water, the *Chlorella* biomass produced during wastewater treatment can also be directly applied in agriculture. For example, *C. vulgaris* biomass was tested as a biofertilizer in wheat germination assays and resulted in a 147% increase in germination index [70]. Biomass of *C. variabilis* generated after wastewater treatment has been studied in the form of aqueous extracts (at 40% and 60% concentrations) for its biofertilizing effect on corn (*Zea mays*) and soybean (*Glycine max*). The results revealed higher mineral levels, antioxidant activity, total phenolic and flavonoid contents, and faster growth rates [73]. However, it is important to note that higher concentrations of a *Chlorella* suspension do not always result in enhanced growth. In some cases, high concentrations can have inhibitory effects. For example, in tomato plants, excessive concentrations of a *Chlorella* suspension negatively affected shoot and root growth [38].

## 7. Commercial *Chlorella*-Based Biofertilizers and Biostimulants

Microalgae-derived agricultural bioproducts based on *Chlorella* are already on the market, although they are still less widespread than those derived from other species. These products are generally commercialized as biostimulants or soil enhancers rather than as conventional fertilizers and are available in various formulations, including dried biomass, liquid extracts, and live-cell suspensions. For example, EnSoil Algae™ is a commercial product based on *Chlorella vulgaris* that illustrates the use of microalgal biomass in agricultural systems [74]. More broadly, *Chlorella* is consistently identified among the microalgal species used at the industrial level to produce biofertilizers, biostimulants, and soil conditioners within a rapidly expanding global market segment [75]. Despite the availability of these products, their large-scale adoption remains limited, primarily due to several technical and economic constraints. These include the high costs associated with large-scale microalgal cultivation, harvesting, and downstream processing, as well as challenges in ensuring product stability and shelf life, particularly for formulations containing labile bioactive compounds or viable cells. In addition, variability in biomass composition and the lack of standardized processing and formulation protocols can result in inconsistent agronomic performance under field conditions, thereby reducing farmer confidence and slowing market uptake.

## 8. Conclusions

The available literature indicates that *Chlorella* species are a promising and versatile source of biofertilizers, capable of enhancing plant growth across a wide range of experimental conditions. Consistently positive effects have been observed from laboratory assays to hydroponic and soil-based systems, including improved seed germination, root development, biomass accumulation, and crop quality. These responses are largely attributed to the presence of bioactive compounds such as phytohormones, amino acids, and micronutrients, which act even at relatively low concentrations. Furthermore, integrating *Chlorella* cultivation with wastewater treatment has emerged as a particularly attractive approach, enabling simultaneous biomass production, nutrient recovery, and water reuse within a circular bioeconomy framework. This dual functionality strengthens the environmental and agronomic value of *Chlorella*-based systems. However, despite these promising results, important limitations remain. Variability in cultivation conditions, biomass processing methods, and application strategies makes it difficult to directly compare studies and define standardized protocols. In addition, most of the available evidence is still derived from controlled conditions, while large-scale field validation remains limited. Overall, current findings support the potential of *Chlorella* as an eco-friendly alternative to chemical fertilizers, but further research is required to optimize production systems, standardize application methods, and assess long-term performance under field conditions.

## Data Availability

The original contributions presented in this study are included in the article material. Further inquiries can be directed to the corresponding authors.
